# Atypical haemolytic uraemic syndrome presenting initially as suspected meningococcal disease: a case report

**DOI:** 10.1186/1752-1947-1-122

**Published:** 2007-10-30

**Authors:** Siddharthan Sivamurthy, John D Mooney, Tom D Kenny

**Affiliations:** 1 Department of Paediatrics, Poole General Hospital, Poole, Dorset, BH15 2JB, UK; 2 Dorset and Somerset Health Protection Unit, Health Protection Agency South West Victoria House Ferndown, Dorset, BH22 9JR, UK

## Abstract

**Background:**

Haemolytic uraemic syndrome (HUS) is the most common cause of acute renal failure in children and is usually linked with Escherichia coli O157 infection. With a fatality rate of around 5%, some reports have associated antibiotic treatment with a worsening prognosis.

**Case Presentation:**

We describe a female infant patient, initially treated for suspected meningococcal septicaemia, who went on to develop renal complications and thrombocytopenia characteristic of HUS. A subsequent positive stool sample for *E. coli *O157 confirmed HUS as an appropriate diagnosis, although there was no evidence of diarrhoea or vomiting throughout the course of her management.

**Conclusion:**

The urgency of early recognition and treatment for suspected meningococcal disease in very young children while entirely appropriate can initially divert attention from other serious conditions. Evidence of infection with *E. coli *O157 infection in this case also highlights what can be a blurred distinction between atypical (non-diarrhoeal) HUS from classical HUS of infective origin.

## Background

Haemolytic uraemic syndrome (HUS) is a predominantly paediatric condition that consists of the simultaneous triad of haemolytic anaemia, thrombocytopenia and acute renal failure. Although very rare (UK incidence: 0.71/100,000/yr [1]), it is the most common cause of acute renal failure in children, has a fatality rate of around 5% and is commonly linked with *Escherichia coli *O157 infection. The potential severity of HUS combined with a worsening outcome associated with some forms of therapy, most notably antibiotics in some reports [2], makes accurate and early diagnosis a critical first step in preventing and reducing severe manifestations of the disease.

## Case Presentation

A 10 month old female infant presented to the family doctor with rhinitis, decreased feeding and non-blanching spots (on her thighs and a few on both cheeks). An upper respiratory tract infection was also reported in the week prior to her illness. There was no evidence of fever, diarrhoea or vomiting at initial presentation. The only child to non-consanguineous parents, she had had an uncomplicated normal term delivery with forceps. There was nothing of significance in her medical history although the couple had experienced the loss of a previous child caused by HELLP (Haemolysis, Elevated Liver enzymes, and Low Platelets syndrome in pregnancy).

On admission to hospital, the infant was alert but pale with a low body temperature of 36.2°C. Cardiovascular signs were: Tachycardia 160–170/min; capillary refill time 2–3 seconds; BP 114/76 mmHg; SaO_2 _99% in air; S1 and S2 normal (no murmurs). Respiratory rate was at the upper range of normal (40/min). CNS evaluation revealed normal anterior fontanelle, no meningeal signs, good tone and power in upper and lower limbs, no focal deficits and no obvious cranial abnormalities. Examination revealed multiple petechial spots on both thighs and a few on her cheeks that had developed since she was seen by her GP an hour earlier. In conjunction with slightly delayed capillary refill and mild tachycardia, the infant was immediately treated as a possible case of meningococcal septicaemia and given iv ceftriaxone and fluid bolus of normal saline. A bolus of HAS (human albumin solution) was administered on the advice of paediatric intensive care (PICU), to be repeated in the event of persistent tachycardia.

Laboratory measurement of red blood cell (RBC) haemoglobin was 83 g/L and red cell indices were within normal ranges [MCV = 73; MCH = 26.8 and MCHC = 363]. White blood cell count and neutrophil counts were above normal (22.4 and 12.1 × 10^9^/L respectively). Platelet count was very depressed at 14 × 10^9^/L (normal range 150–400 × 10^9^/L). Blood metabolic indicators were pH = 7.323, pCO_2 _= 5.28; BE = -5.0; HCO_3 _= 20.0 mmol/L; lactate = 1.3 mmol/L.

Biochemical analysis showed normal plasma values for sodium (140 mmol/L) potassium (5.1 mmol/L) and creatinine (53 μmol/L) but raised urea (16.2 mmol/L: normal range 2.5–6.4) and raised alkaline phosphatase (ALP = 2537 iu/L; normal range: 40-130). C reactive protein (8 mg/L) was also slightly elevated. From the clinical presentation and laboratory findings the presumptive diagnosis was haemolytic uraemic syndrome (HUS). A subsequent blood film showing fragmented RBCs together with increased polychromasia and thrombocytopenia, confirmed HUS as the most likely underlying pathology, and the patient was transferred to paediatric nephrology at a tertiary centre in case of the need for dialysis. Blood cultures over the next 2 days were negative, as was PCR for *Neisseria Meningitidis*. A stool sample returned positive for *E. coli *O157, consistent with a diagnosis of HUS secondary to *E. coli *infection. The subsequent administration of a standard *E. coli *trawling questionnaire by the local health protection team revealed no notable risk factors for gastroenteritis (i.e. zoo visits/contact with animals or symptoms in family members). The patient was managed conservatively at the specialist centre without the need for dialysis. She received one packed red cell transfusion and urea monitoring showed no rise. A spontaneous increase in her platelet count was noted. Follow up blood testing after one week showed increased Hb. She was managed as an inpatient for one week and is currently being followed as an outpatient.

## Discussion and Conclusion

The severity of meningococcal septicaemia and its frequent non-specific presentation means that a high index of clinical suspicion must always be maintained for any infant with a non-blanching petechial rash. Infants with meningococcal infection are also likely to have a capillary refill time of more than 2 seconds (as was the case with this patient) and can be apyrexial [3]. Although it would typically be expected that a meningococcal presentation would include an unambiguous neutrophilia and a higher C reactive protein [3], a first impression of meningococcal disease on the basis of a rapidly spreading rash and reduced capillary refill time was appropriate at initial assessment.

This particular case is further complicated by likely classification as 'atypical' HUS on the basis of her non-diarrhoeal presentation [4]. Most commonly a secondary complication of intestinal infection with verotoxigenic *E. coli *O157 (or V-TEC), HUS is usually preceded by a diarrhoeal episode which was absent in this case. The later laboratory results revealing a pronounced thrombocytopenia are more characteristic of HUS, as is the very low red blood cell Hb and highly elevated ALP, indicating liver involvement. HUS is also usually associated with raised creatinine levels, which remained at the lower end of the normal range for the duration of this patient's supervision. The evidence of ruptured red blood cells however was sufficient to change the focus of treatment to likely HUS and the subsequent isolation of *E. coli *O157 confirmed this as an appropriate clinical decision.

Among the factors which have been linked with a worsening prognosis of HUS, which can be fatal in 5 – 10% of cases, is pre-treatment with antibiotics [2]. The early treatment of this case as a possible meningococcal septicaemia, while entirely appropriate given the clinical presentation, may well have exacerbated the course of her illness. While the authors would certainly not advocate reducing vigilance to the possibility of meningococcal disease in young children with a petechial rash, it remains important to maintain an open verdict if all the signs are not consistent. This is especially apparent in the case of an atypical presentation of HUS which, although rare, is an equally serious and life threatening disease and delayed recognition or inappropriate treatment can significantly worsen the outcome.

Finally it is still uncertain as to how this case should be classified. Several authors have made the distinction that non-diarrhoeal HUS should be considered as 'atypical' with a distinct pathology unrelated to bacterial infection and a genetic component involving complement factors [5]. Strictly speaking therefore, the presence of *E. coli *O157 should exclude this case as 'atypical HUS'. The distinction is an important one from the point of view of the patient, since non-typical HUS cases are susceptible to recurrence. The loss of a previous child in this case with the development of HELLP syndrome in pregnancy, is also suggestive of a genetic predisposition however since it shares many of the clinical features of HUS [6].

## Competing interests

The author(s) declare that they have no competing interests.

## Authors' contributions

SS was the lead physician responsible for the clinical care of the case, supplied all clinical details and helped revise the draft report to completion.

JDM jointly conceived of the idea for the article (with TDK), searched the background literature, wrote the first draft and refined the text in accordance with comments from SS and TDK.

TDK received initial notification of the case, advised on the health protection response, advised on clinical interpretation of the presenting details and assisted in the drafting of the paper.
